# Adalimumab Induced or Provoked MS in Patient with Autoimmune Uveitis: A Case Report and Review of the Literature

**DOI:** 10.1155/2016/1423131

**Published:** 2016-10-20

**Authors:** Rana Alnasser Alsukhni, Ziena Jriekh, Yasmin Aboras

**Affiliations:** ^1^Division of Neurology, Department of Internal Medicine, Aleppo University Hospital, Aleppo, Syria; ^2^Division of Rheumatology, Department of Internal Medicine, Aleppo University Hospital, Aleppo, Syria

## Abstract

Anti-tumor necrosis factor *α* (anti-TNF-*α*) agents have been widely used in the field of autoimmune diseases and have proved decisive efficacy and relative safety. Data concerning their adverse effects has been lately describing central nervous system (CNS) demyelination process at escalating basis.* Case Presentation*. A 23-year-old male with autoimmune uveitis and a family history of multiple sclerosis (MS) developed two neurological attacks, after Adalimumab infusion, simultaneously with several cerebral lesions on magnetic resonance imaging (MRI). Hence the diagnosis of Adalimumab induced MS was suspected.* Conclusion*. This case is reported to tell physicians to be cautious when using anti-TNF-*α* in patients with family history of MS and to reconsider the risk of MS in patients with autoimmune diseases.

## 1. Introduction

TNF-*α* is a well-known proinflammatory and immunoregulatory cytokine. It mediates inflammatory cell recruitment to the site of the infection by activating endothelial cells. Elevated levels of this cytokine, however, were detected both locally and systemically in patients with inflammatory disorders like arthritis [1]. Drugs that block the effect of TNF-*α* have shown beyond dispute role in modulating the activity of many autoimmune diseases. Depending on their mechanism of action, anti-TNF-*α* drugs are divided into monoclonal antibodies (Infliximab, Adalimumab, Golimumab, and Certolizumab) and soluble TNF-*α* receptors (Etanercept). Many side effects have been recorded during the relatively short history of utilizing anti-TNF-*α* in the field of autoimmune diseases such as infectious disease [2], autoimmune disease [3, 4], blood dyscrasia [5, 6], pulmonary fibrosis [7], congestive heart failure [8], and malignancies [9] among other adverse effects such as central and peripheral demyelination of the nervous system and optic neuritis.

CNS demyelination was conducted by several reports suggesting triggering or exacerbating demyelination diseases induced by treatment with anti-TNF-*α*.

In Table 1 we review the most important studies in medical literature concerning this idea.

## 2. Case Presentation

**Figure 1:**
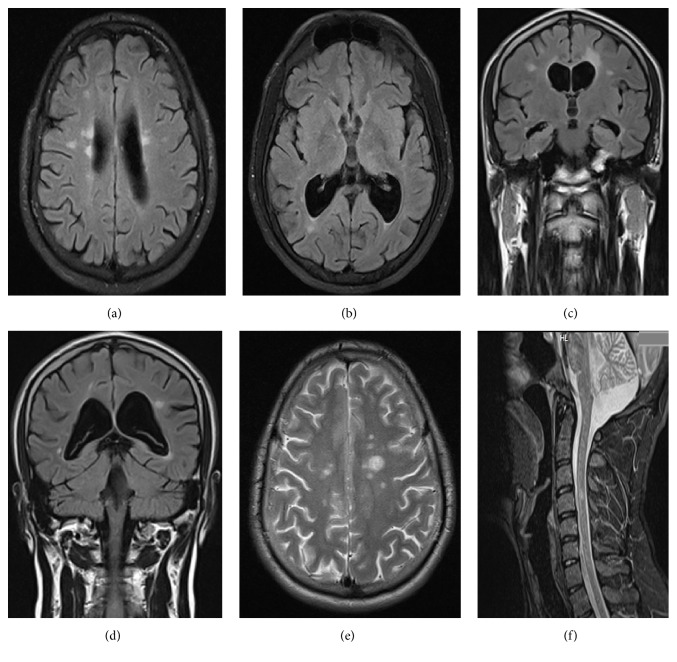
(a, b, c, and d) Flair MRI slices (axial and coronal slices) show periventricular and juxtacortical high intensity lesions. (e) Axial T2 MRI shows multiple juxtacortical high intensity lesions. (f) Sagittal T2 MRI of the cervical spinal cord shows T2 hyperintense lesion at the level of C4.

A 23-year-old Arabic gentleman from Syria had a positive family history of MS affecting two of his uncles (maternal and paternal uncles; no blood relationship existed between the parents) and idiopathic epilepsy affecting his sister. His past medical history was positive for chronic autoimmune uveitis since the age of 13. It was then treated with Methotrexate with poor response, and thus it was substituted for combination of Cyclosporine and Azathioprine which achieved a better control. A year ago, Azathioprine was suspended for a while because of the political situation and Adalimumab was initiated. Few days following the second dose of Adalimumab, the patient developed right hemibody anesthesia which lasted two weeks and was successfully treated with intravenous methylprednisolone. Adalimumab was then stopped and both of Azathioprine at a dose of 3 mg/kg and oral prednisolone were initiated. Five months later, brain magnetic resonance imaging (MRI) was requested and revealed multiple periventricular and juxtacortical lesions with high T2 signal intensity along with T2 hypersignal lesion in the spinal cord at the level of the fourth cervical vertebra C4; none of these lesions were contrast enhancing (Figure 1).

Visually evoked potentials (VEP) were also performed and were normal. Cerebrospinal fluid (CSF) analysis was normal and negative for oligoclonal bands (OCBs).

Eight months following Adalimumab infusion, the patient had right lower limb paresis which was also successfully treated with methylprednisolone, and normal strength was restored within three weeks. Six months after then, the patient was referred by a rheumatologist to a neurology clinic for consultation and followup though there has been no new attack since then.

**Figure 2:**
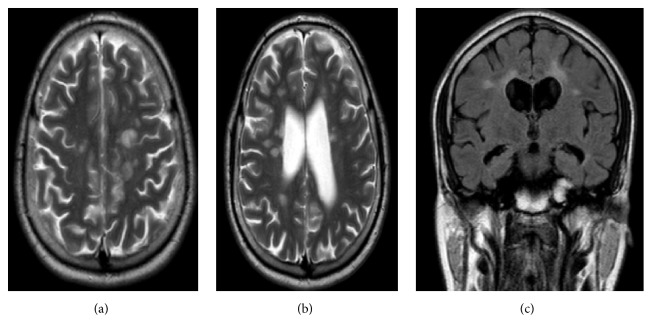
(a and b) Axial T2 MRI shows multiple periventricular and juxtacortical high intensity lesions. (c) Coronal flair MRI shows periventricular and juxtacortical high intensity lesions.

Neurological examination was normal except for anisocoria with slightly irregular pupils and bilaterally sluggish reaction to light (an abnormality that was explained by chronic uveitis) with normal fundoscopy and visual acuity. Mild right facial and body hemihypoesthesia, absent abdominal reflexes over the right side, generalized hyperreflexia (+3), and mild right lower limb spasticity were also noticed. To find out the relationship between the uveitis and the neurological attacks, the type of uveitis was reassessed and revealed hints of anterior and intermediate uveitis with no signs of active inflammation. An X-ray of sacroiliac joints and chest X-ray were then requested to rule out both subclinical ankylosing spondylitis and sarcoidosis and were both normal. Brain MRI with contrast was repeated and revealed no new lesions comparing with the previous one (Figure 2).

The previous laboratory study was reviewed and showed normal ordinary labs including erythrocyte sedimentation rate (ESR) and C-reactive protein (CRP). Antinuclear antibodies (ANA), rheumatic factor (RF), and antineutrophil cytoplasmic antibodies C-ANCA and P-ANCA were all negative. Human leukocyte antigen (HLA) analysis revealed HLA-B27. CSF analysis showed acellular fluid with immunoglobulin G (IgG) index of 0.53, normal protein, lactate dehydrogenase (LDH), and glucose levels. Despite the lack of enhancement, diagnosis of Adalimumab induced MS was suspected in this patient with HLAB-27 associated uveitis depending on Mcdonald's criteria for space and time distribution and the temporal relationship between the first neurological attack and drug initiation besides the positive family history of MS. Since there were no active lesions on MRI, treatment plan was continued with no modification and regular clinical and radiological followup was suggested.

## 3. Discussion

This case describes two separate neurological attacks in a young patient with HLA-B27 associated uveitis and a family history of multiple sclerosis. Although neurological attacks meet Mcdonald's criteria for time and space distribution, many differential diagnoses are plausible for this presentation, MS with associated uveitis, HLA-B27 associated uveitis and a newly diagnosed MS as two separate entities, and systemic vasculitis induced uveitis and cerebral lesions. Infectious causes and lymphoma, however, are improbable due to the prolonged history and negative laboratory investigations.

A certain type of uveitis, uveitis pars planitis, is considered a manifestation of MS that often precedes or, less often, accompanies the first attacks [17]. The type of uveitis was reassessed accordingly and revealed both anterior and intermediate uveitis which is, along with the prolonged history of uveitis, less compatible with MS and more compatible with HLA-B27 uveitis with or without seronegative arthritis such as ankylosing spondylitis, psoriasis, and inflammatory bowel disease (IBD). During 10 years of uveitis, the patient had no arthritis, gastrointestinal symptoms, skin lesions, or even lower back pain. Moreover, AXR of the pelvis revealed no sclerotic changes.

Despite its obscurity, a relationship between MS and autoimmune diseases was deduced because of the high prevalence of MS in patients with autoimmune diseases as suggested by many reports [18–20]. This speculation added to the positive family history of MS renders this patient highly susceptible to MS. Furthermore, the temporal relationship between the first attack and Adalimumab infusion makes Adalimumab induced or provoked MS the most likely diagnosis.

Suspected MS following anti-TNF-*α* treatment in patients with family history of MS was reported in many cases [14, 18] which recommended that anti-TNF-*α* should be avoided in such patients and advocated requesting cerebral MRI before treatment initiation in patients with high risk of MS [21, 22].

Several mechanisms have been proposed to interpret the paradox of MS activation in spite of amelioration of systemic inflammation following anti-TNF-*α* infusion such as the presence of blood brain barrier (BBB). This barrier halts the entry of anti-TNF-*α* drugs to CNS. Meanwhile, these drugs enhance T cell receptor signaling and the functionality of antigen presenting cells and, consequently, they activate CNS demyelination. This is the essence of lack of entry theory. Presence of BBB was also the culprit in sponge theory wherein TNF-*α* is supposed to be drawn out of joint synovial fluid following TNF-*α* antagonism whereas BBB prevents this process in CNS. Thus TNF-*α* gradient develops across BBB and results in upregulation of TNF-*α* receptor expression in CNS. Moreover, severely destructed BBB in experimental autoimmune encephalopathy mice models besides the high dose used have been supposed to facilitate anti-TNF-*α* entry to CNS and permit the subsequent amelioration [23]. Other theories hypothesized that tumor necrosis factor receptor 2 (TNFR2) is a potential regenerator of oligodendrocytes. Hence blocking TNF-*α* may prevent this natural mechanism and suppress remyelination [24]. Another hypothesis postulated that anti-TNF-*α* drugs promote interferon *γ* (INF-*γ*) expression and this in turn disturbs the balance between INF-*β* and INF-*γ* levels which triggers demyelination process [23]. Whether the precise mechanism is uncovering or inducing demyelination is still undetermined.

Although some points argue against MS such as OCBs negativity and the lack of contrast enhancement, MS negative OCBs are a well-established entity that represents 20–30% of MS patients [25]. Moreover, OCBs could be detected later on during the circumstance of MS. No clear data is available regarding frequency of OCBs negativity in anti-TNF-*α* induced MS since most of the reports of similar cases did not mention the results of OCBs analysis. The lack of enhancement, however, may be explained by the time window between performing MRI and the clinical attacks; such window would have guaranteed the resolution of enhancement if it existed in addition to treatment with Azathioprine, a drug that has been accepted as a disease modifying treatment of MS [26].

Asymptomatic cerebral lesions highly suggestive of MS are widely described in patients with family history of MS under the title of radiographically isolated syndrome (RIS) [27, 28], but explicit clinical attacks turn RIS into clinically isolated syndrome (CIS) or MS. Most of the reported cases depicted several months of using anti-TNF-*α* agents preceding the development of clinical attacks [13, 16], while in this case development of the first clinical attack took place days following the second dose, a period that suggests a process of uncovering latent MS rather than inducing it. Even though second clinical attack occurred approximately eight months after halting Adalimumab, MRI performed six months later revealed no active disease; consequently no treatment modification was suggested, and if hints of clinical or radiological activity are noticed later on, Rituximab would be the treatment of choice of both uveitis and MS.

## 4. Conclusion

MS is highly expected in patients with family history of MS and, to a lesser degree, in patients with autoimmune diseases, and physician should consider such risks in patients who are eligible for anti-TNF treatment by detailed history, examination, and close followup as well as performing MRI prior to therapy initiation in highly suspected individuals.

## Figures and Tables

**Table 1:** 

Date	Authors	Title	Summary
1994	Baker et al.	“Control of Established Experimental Allergic Encephalomyelitis by Inhibition of Tumor Necrosis Factor (TNF) Activity within the Central Nervous System Using Monoclonal Antibodies and TNF Receptor-Immunoglobulin Fusion Proteins” [10]	They suggested that anti-TNF-*α* were effective in ameliorating demyelination process in several transgenic mouse models of experimental autoimmune encephalomyelitis.
1995	Selmaj et al.	“Prevention of Chronic Relapsing Experimental Autoimmune Encephalomyelitis by Soluble Tumor Necrosis Factor Receptor” [11]	

1996	Van Oosten et al.	“Increased MRI Activity and Immune Activation in Two Multiple Sclerosis Patients Treated with the Monoclonal Anti-Tumor Necrosis Factor Antibody cA2” [12]	It reported both radiological and laboratory deterioration in two patients with rapidly progressive MS treated with intravenous infusions of a humanized mouse monoclonal TNF-*α* antibody.

2001	Mohan et al.	“Demyelination Occurring during Anti-Tumor Necrosis Factors *α* Therapy for Inflammatory Arthritides” [13]	It conducted cases of 19 patients with neurologic events suggestive of demyelination following either Etanercept or Infliximab infusion. These cases were identified by searching of the AERS database.

2013	Andreadou et al.	“Demyelinating Disease following Anti-TNFa Treatment: A Causal or Coincidental Association?” [14]	It reported four cases of suspected MS after treatment with anti-TNF-*α*.

2013	Seror et al.	“Pattern of Demyelination Occurring during Anti-TNF-*α* Therapy: A French National Survey” [15]	A research about the pattern of demyelination in 33 patients developing demyelinating disorders after treatment with anti-TNF-*α*.

2014	Kaltsonoudis et al.	“Neurological Adverse Events in Patients Receiving Anti-TNF Therapy: A Prospective Imaging and Electrophysiological Study” [16]	A study included 75 patients who were treated with anti-TNF-*α*. These patients were followed up for an average period of 13 months and three of them developed neurological symptoms; single patient had CNS demyelination lesions, another one had optic neuritis, and the third patient developed peripheral demyelination.

## Abbreviations

TNF-*α*: Tumor necrosis factor *α*
CNS: Central nervous system
MS: Multiple sclerosis
MRI: Magnetic resonance imaging
VEP: Visually evoked potentials
CSF: Cerebrospinal fluid
OCBs: Oligoclonal bands
ESR: Erythrocyte sedimentation rate
CRP: C-reactive protein
ANA: Antinuclear antibodies
RF: Rheumatic factor
ANCA: Antineutrophil cytoplasmic antibodies
HLA: Human leukocyte antigen
IgG: Immunoglobulin G
LDH: Lactate dehydrogenase
IBD: Inflammatory bowel disease
BBB: Blood brain barrier
TNFR2: Tumor necrosis factor receptor 2
INF: Interferon
RIS: Radiographically isolated syndrome
CIS: Clinically isolated syndrome.

## Additional Points

*Availability of Data and Materials.* All available data are included within the manuscript.
